# Radiation‐Related Knowledge and Attitudes Toward Medical Imaging Among the General Population in Iraq: A Cross‐Sectional Survey

**DOI:** 10.1002/jmrs.70123

**Published:** 2026-09-30

**Authors:** Walid Al‐Qerem, Aiham Assie Hammood, Alaa Hussein Alsajri, Anan Jarab, Judith Eberhardt, Lama Sawaftah

**Affiliations:** ^1^ Department of Pharmacy, Faculty of Pharmacy Al‐Zaytoonah University of Jordan Amman Jordan; ^2^ Department of Physics, College of Science University of Tikrit Tikrit Iraq; ^3^ Department of Pharmacy Al‐Rasheed University College Baghdad Iraq; ^4^ Department of Clinical Pharmacy, Faculty of Pharmacy Jordan University of Science and Technology Irbid Jordan; ^5^ Department of Psychology, School of Social Sciences, Humanities and Law Teesside University Middlesbrough UK

**Keywords:** cross‐sectional survey, medical imaging, patient education, public knowledge, radiation safety, radiography

## Abstract

**Introduction:**

Medical imaging is widely used in Iraq, but evidence regarding public knowledge of radiation‐related risks and attitudes toward radiation safety and communication is limited. This study assessed radiation‐related knowledge, attitudes, safety practices, and information provision among adults in Iraq, and examined associated factors.

**Methods:**

A cross‐sectional online convenience survey was conducted among 1274 adults in Iraq. Participants were recruited through a Google Forms link distributed via Facebook, WhatsApp, Viber, and Telegram. The questionnaire assessed demographics, previous radiation exposure, radiation‐related knowledge, attitudes toward radiation safety, and information‐seeking practices. Knowledge and attitude scores were analysed using descriptive statistics and quantile regression.

**Results:**

The sample was evenly distributed by sex and was predominantly aged 18–30 years (*n* = 786, 61.7%) and urban residents (*n* = 830, 65.1%). Median knowledge was 5/10 (IQR: 3–7) and median attitude was 17/25 (IQR: 15–18). Most participants recognized cancer and reproductive risks associated with radiation exposure, but important knowledge gaps remained regarding CT dose equivalence and the non‐ionizing nature of MRI. Participants generally reported cautious attitudes toward repeated radiation exposure and positive views toward radiation‐safety education, although perceptions of warning signs and direct communication with healthcare professionals were less favourable. No demographic, educational, work/study‐field, residence, or radiation‐exposure variable was significantly associated with knowledge in the adjusted model, whereas a higher knowledge score and female gender were associated with higher attitude scores.

**Conclusions:**

Substantial gaps in radiation‐related understanding persist despite widespread imaging use. Attitudes toward radiation safety were generally cautious and supportive of education, but direct engagement with healthcare professionals was limited. Structured patient education and clearer communication about imaging benefits, risks, and safety measures may improve radiation literacy and informed attitudes toward medical imaging.

## Graphical abstract

A cross‐sectional survey of 1274 adults residing in Iraq found a median radiation‐related knowledge score of 5/10 and a median attitude score of 17/25. Only 18% correctly identified that MRI does not use ionizing radiation, while 36% recognized that a spiral CT scan may involve radiation equivalent to 100–800 conventional X‐rays. Median quantile regression showed that higher knowledge scores and female sex were associated with higher attitude scores. These findings highlight specific gaps in radiation‐related understanding and support the integration of clear, structured radiation‐risk education into routine imaging care.

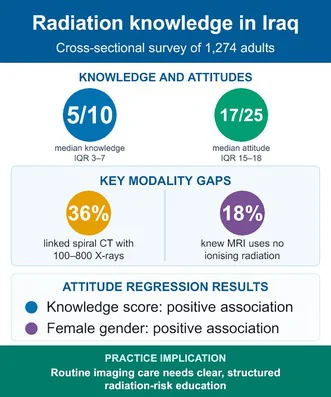

## 1 Introduction

Radiation exposure can have a range of harmful effects on the human body. Many medical imaging procedures, including conventional X‐ray imaging and computed tomography (CT), are widely used for diagnosis, disease monitoring, and clinical decision‐making and involve exposure to ionizing radiation [1]. Despite their important clinical benefits, ionizing radiation carries inherent risks due to its ability to damage biological tissues and alter cellular DNA, which may lead to both short‐ and long‐term health consequences [2]. These effects depend on several factors, including radiation dose and frequency, age, and tissue sensitivity, with high or repeated exposures associated with an increased risk of malignancies, cataracts, infertility, impaired growth in children, and adverse fetal outcomes during pregnancy [3, 4, 5]. CT imaging, in particular, delivers substantially higher radiation doses than standard radiography and is a major contributor to population exposure from medical imaging worldwide [6].

Despite advances in radiation protection principles, including justification, optimisation, and the “as low as reasonably achievable” (ALARA) concept [7], public awareness and understanding of radiation risks remain limited [8]. Insufficient patient knowledge may contribute to unnecessary anxiety, poor decision‐making, or acceptance of imaging procedures that are not clinically justified. Conversely, underestimating radiation risks may lead to repeated or unnecessary exposure, increasing cumulative dose and long‐term health risks [9, 10].

Previous studies have identified gaps in knowledge regarding radiation sources, relative radiation doses, and the distinction between ionizing and non‐ionizing imaging modalities [11, 12]. Attitudes toward radiation are influenced by trust in healthcare professionals, the perceived benefits of imaging, and access to clear information about potential risks and safety measures [13], making patient education an important component of radiation‐safety practice [14]. A recent cross‐sectional study among dental professionals in Jordan similarly reported limited knowledge and suboptimal radiation‐safety practices [15]. In Iraq, available research has focused mainly on medical students [16], physicians, and other personnel working in radiology departments [17, 18]. Although these studies provide useful evidence from selected professional groups, their findings cannot be generalised to the wider adult population and do not adequately describe public knowledge, attitudes toward radiation risks and safety practices, or information‐seeking behaviour. Addressing this gap is important because adults frequently undergo or participate in decisions concerning diagnostic imaging procedures across different healthcare settings.

Given the widespread use of medical imaging and the potential health implications of radiation exposure, assessing public knowledge, attitudes toward radiation risks, safety practices, and communication about medical imaging is important. Such assessments can identify knowledge gaps, inform targeted educational interventions, and support strategies to improve patient safety. Accordingly, this study aimed to assess radiation‐related knowledge and attitudes toward radiation risks, safety practices, and communication about medical imaging among adults residing in Iraq and to identify factors associated with these outcomes.

## 2 Method

### 2.1 Study Design and Setting

This study used a cross‐sectional online survey design and was conducted across multiple regions of Iraq. Data were collected between September and December 2025 using a structured questionnaire developed in Google Forms, with the survey link distributed via widely used social media platforms, including Facebook, WhatsApp, Viber, and Telegram. These platforms were selected due to their widespread use in Iraq and their ability to reach a diverse population. According to the 2024 national census, Iraq has an estimated population of 45.4 million, with a near‐equal gender distribution (50.1% male and 49.9% female) [19]. This demographic context provided a reference point for assessing the representativeness of the study sample.

### 2.2 Study Population and Sampling

The study targeted individuals aged 18 years or older who were residing in Iraq and able to read and understand Arabic. Given the online format and voluntary participation, a convenience sampling approach was used. Participants were required to provide electronic informed consent before completing the questionnaire. Exclusion criteria were age under 18 years, residence outside Iraq, and duplicate submissions. All questionnaire items required for analysis were configured as mandatory. Participants could leave the survey at any time before submission, but Google Forms did not permit submission until all mandatory items had been completed. To minimise duplicate submissions, Google Forms was configured to limit participation to one response per logged Google account. Consequently, all submitted questionnaires were complete, and the final dataset contained no missing values.

### 2.3 Data Collection Tool

Data were collected using a structured questionnaire adapted from two previously published studies conducted in Saudi Arabia [8] and Jordan [20]. The instrument was modified to reflect the Iraqi context. The final questionnaire consisted of five sections: Demographic characteristics, radiation exposure experience, radiation risk awareness, attitudes toward radiation safety, and information sources and practices. Radiation‐related knowledge was assessed using 10 true‐or‐false items. One point was awarded for each correct response and zero points for an incorrect response, resulting in a total score ranging from 0 to 10. No validated cut‐off points were available for qualitative classification; therefore, the total score was analysed as a continuous variable. Attitudes were assessed using five items measured on a five‐point Likert scale, producing a total score ranging from 5 to 25. Negatively worded items were reverse‐coded before calculating the total scores. Practice‐related items examined prior exposure to educational materials, reading of radiation warning signs, and communication with healthcare providers. The knowledge and attitude totals were developed as content‐based composite indices rather than as reflective psychometric scales intended to measure a single latent construct or a predefined multidimensional domain structure. The knowledge items sampled distinct factual aspects of radiation and medical imaging, while the attitude items represented several prespecified views related to radiation risk, safety, communication, and education. Accordingly, factor‐analytic assessment of structural validity was not prespecified. The resulting totals should be interpreted as summary indices of the included items rather than as validated latent‐trait measures.

Before translation, content validity was assessed by a multidisciplinary panel of five experts comprising three specialists in radiology and two specialists in public health. Using a structured review based on established content‐validity guidance, the panel evaluated the relevance of each item to the intended constructs and study population, the comprehensiveness of domain coverage, the clarity and comprehensibility of the wording, the appropriateness of the response options, and cultural suitability for use in Iraq. The panel's recommendations were consolidated by the research team, and agreed revisions were incorporated before the linguistic‐adaptation stage [21, 22].

The content‐reviewed English questionnaire then underwent a structured forward‐ and back‐translation process in accordance with established cross‐cultural adaptation guidance [23, 24, 25]. Two translators independently produced English‐to‐Arabic forward translations, which were compared and reconciled into a single preliminary Arabic version. Two additional translators, who had not participated in the forward‐translation stage and were blind to the original English questionnaire, then independently back‐translated the reconciled Arabic version into English, resulting in four translators participating across the two translation directions. The original English questionnaire, both forward translations, the reconciled Arabic version, and both back‐translations were jointly reviewed by the four translators and the research team. Differences in conceptual meaning, terminology, clarity, and cultural appropriateness were discussed and resolved to produce a pre‐final Arabic version.

Participants for cognitive pretesting were recruited using purposive maximum‐variation sampling from Iraqi adults who satisfied the eligibility criteria for the main study. A brief screening form was used to identify potential participants according to age, sex, educational attainment, geographic location, urban or rural residence, and familiarity with online questionnaires. Selection was guided by a sampling matrix to ensure the inclusion of participants with diverse characteristics, with particular emphasis on individuals who might experience difficulty interpreting formal or health‐related terminology. Cognitive pretesting was conducted in two iterative rounds, with 15 participants in each round. Participants were approached individually in person by the researchers and informed that the purpose was to evaluate the questionnaire rather than their knowledge or abilities. During individual cognitive interviews, participants completed the questionnaire and responded to neutral verbal probes examining item comprehension, interpretation, recall, response selection, cultural appropriateness, and the clarity of the response options. Findings were reviewed after each round, and only minimal language changes were required.

Following cognitive pretesting and revision, the online questionnaire underwent technical pilot testing among a separate purposively selected sample of 30 eligible Iraqi adults. Participants were selected to provide variation in age, education, place of residence, device type, and internet connectivity. The pilot was conducted as a closed soft launch using the same recruitment information, eligibility screening, electronic consent procedures, survey platform, and questionnaire configuration intended for the main study. Participants completed the questionnaire independently and subsequently provided structured feedback regarding navigation, item display, font readability, response selection, completion time, data submission, and any technical difficulties. Pilot responses were not incorporated into the final analytical dataset [23, 24].

### 2.4 Ethical Considerations

Ethical approval for this study was granted by the Department of Pharmacy at Al‐Rasheed University College, reference number: RUCPD10012604. The study was conducted in accordance with the ethical principles outlined in the Declaration of Helsinki. Participation was voluntary, and electronic informed consent was obtained from all participants prior to questionnaire completion. No personally identifiable information was collected, and participants were informed that they could withdraw at any time before submitting their responses. Because no personally identifiable information was collected and responses were anonymised upon submission, it was not possible to identify, withdraw, or delete an individual response after submission. All data were stored securely and used solely for research purposes.

### 2.5 Sample Size Calculation

This study employed a cross‐sectional online survey design and used convenience sampling as the participant‐recruitment method. The cross‐sectional design enabled data to be collected from participants at a single point in time, while convenience sampling was used because the questionnaire was distributed voluntarily through online social media platforms. The required sample size for this study was calculated using the Krejcie and Morgan method [26], which is based on the formula for estimating sample size from a given population at a 95% confidence level with a 5% margin of error. Based on the estimated adult population in Iraq, the minimum required sample size was determined to be 385 participants. The study recruited 1274 participants, exceeding the minimum required sample size and improving the precision of the estimates and the stability of the multivariable analyses.

### 2.6 Statistical Analysis

Data were analysed using IBM SPSS Statistics (version 26). Normality was assessed using the Kolmogorov–Smirnov test, which indicated non‐normal distributions; therefore, nonparametric methods were applied. Descriptive statistics were used to summarise study variables, with continuous variables reported as medians and interquartile ranges (IQRs) and categorical variables presented as frequencies and percentages. The final analytical dataset contained no missing values; therefore, no imputation or case‐wise exclusions for missing data were required.

Knowledge scores were calculated by assigning one point for each correct response and zero points for incorrect responses. Negatively worded items were reverse‐coded before score calculation. Attitudes toward radiation‐related risks were assessed using a five‐item composite measured on a five‐point Likert scale ranging from strongly disagree (1) to strongly agree (5), yielding a maximum possible score of 25. Negatively worded attitude items were reverse‐coded before analysis. Internal consistency of the knowledge and attitude composites was assessed using Cronbach's alpha. Reliability coefficients were not calculated for the demographic, exposure, information‐source, or practice sections because these comprised standalone items rather than internally homogeneous multi‐item scales.

Median quantile regression at the 0.50 quantile was used to examine adjusted associations with knowledge and attitude scores. The knowledge model included gender, age group, educational level, work/study field, area of residence, continuous occupational radiation exposure, and direct radiation exposure during the previous six months. The attitude model included the same covariates together with knowledge score, which was interpreted as an adjusted cross‐sectional association rather than a causal effect. This approach was chosen because both scores were bounded and non‐normally distributed, and the primary summary measure was the median. Quantile regression estimates changes in the conditional median and does not require normally distributed residuals. It also permits simultaneous adjustment for multiple covariates, unlike unadjusted nonparametric group‐comparison tests. Multicollinearity was assessed using variance inflation factors (VIFs), with all values below 3, indicating no evidence of collinearity. Statistical significance was set at *p* < 0.05.

## 3 Results

The study included 1274 participants, with sociodemographic characteristics summarised in Table 1. The sample was nearly evenly distributed by sex, comprising 645 female participants (50.6%) and 629 male participants (49.4%). Most respondents were aged 18–30 years (*n* = 786, 61.7%), followed by those aged 31–40 years (*n* = 302, 23.7%), while 186 participants (14.6%) were older than 40 years. More than half of the participants held a bachelor's degree (*n* = 690, 54.2%), and approximately one quarter had completed postgraduate education (*n* = 326, 25.6%). In terms of work or field of study, 686 participants (53.8%) had non‐medical backgrounds, 326 (25.6%) were in medical fields, and 262 (20.6%) worked or studied in radiation‐related fields. Most participants resided in urban areas (*n* = 830, 65.1%), and 1096 participants (86.0%) reported having no chronic disease.

**TABLE 1:** Sociodemographic characteristics of the study sample.

Characteristic	Category	Count (%)
Gender	Female	645 (50.6%)
	Male	629 (49.4%)
Age	18–30 years	786 (61.7%)
	31–40 years	302 (23.7%)
	Older than 40 years	186 (14.6%)
Educational level	Primary/secondary school or less	126 (9.9%)
	Diploma	132 (10.4%)
	Bachelor's degree	690 (54.2%)
	Postgraduate degree	326 (25.6%)
Work/study field	Radiation‐related field (radiographer, radiologist, or medical physicist)	262 (20.6%)
	Medical field	326 (25.6%)
	Non‐medical field	686 (53.8%)
Area of residence	Rural	444 (34.9%)
	Urban	830 (65.1%)
Presence of chronic diseases	No	1096 (86%)
	Yes	178 (14%)

In terms of radiation exposure and experience, 124 participants (9.7%) reported continuous occupational exposure to radiation, 892 (70.0%) reported no such exposure, and 258 (20.3%) were unsure. Nearly half of the participants, 630 (49.5%), indicated that their most recent X‐ray examination was performed following a physician's order, whereas 155 (12.2%) reported undergoing imaging as part of a personal check‐up and 489 (38.4%) reported no prior radiological imaging. Regarding information provision, 648 participants (50.9%) reported being informed by a radiographer about radiation risks, whereas 626 (49.1%) reported that they had not been informed. Similarly, 664 participants (52.1%) reported receiving information about necessary precautions during radiation exposure, while 610 (47.9%) reported that they had not. Patterns of direct radiation exposure during the previous six months and use of protective equipment are shown in Figure 1 and Figure 2.

**FIGURE 1:**
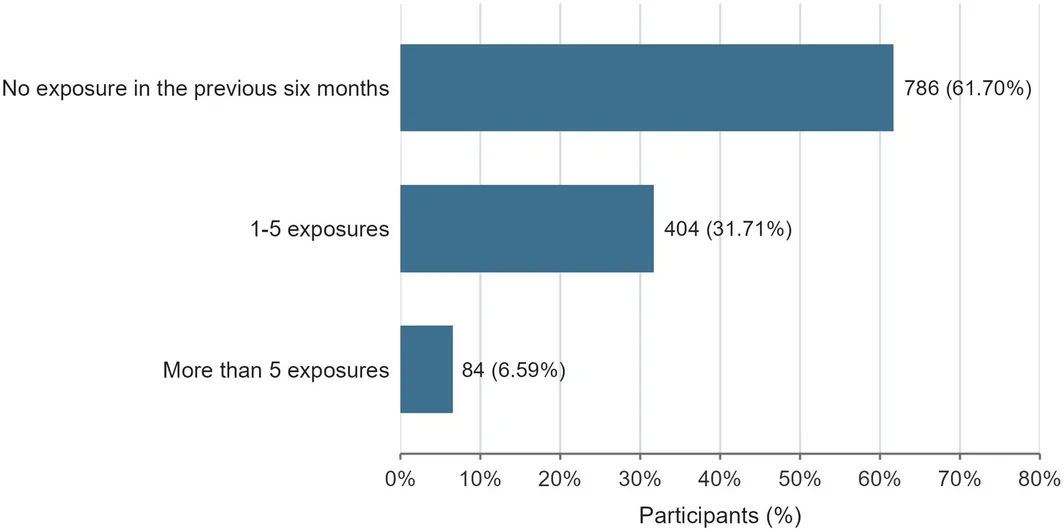
Frequency of self‐reported direct radiation exposure during the previous six months among surveyed adults in Iraq (*N* = 1,274). Bars show the number and percentage of participants in each exposure category.

**FIGURE 2:**
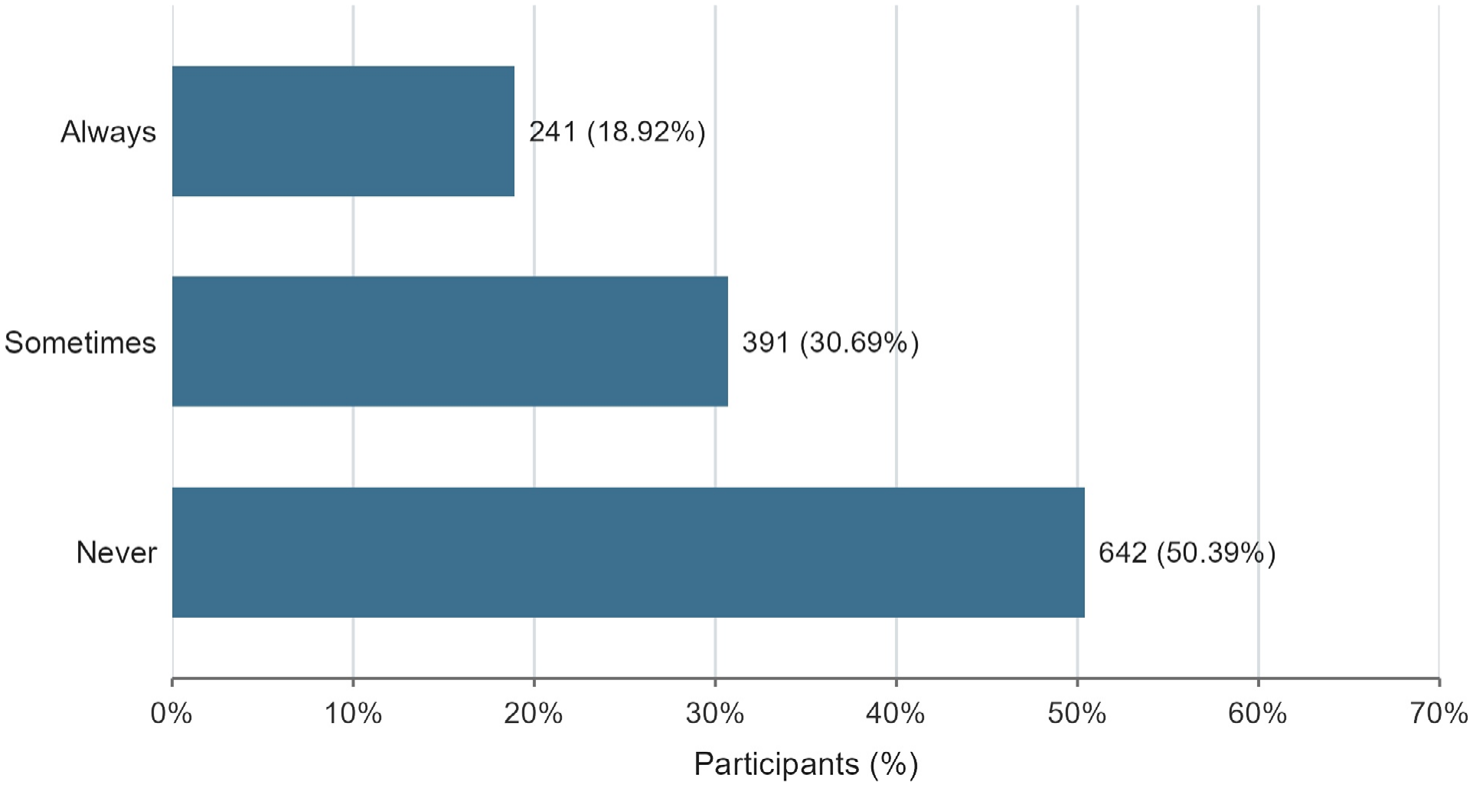
Self‐reported use of protective equipment during radiation exposure among surveyed adults in Iraq (*N* = 1,274). Bars show the frequency and percentage of participants reporting always, sometimes, or never using protective equipment.

Assessment of radiation‐related knowledge showed considerable variation across individual items (Table S1). Most participants correctly identified that radiation can cause cancer (*n* = 1,031, 80.9%), that high doses of radiation may result in temporary or permanent infertility (*n* = 822, 64.5%), and that exposure during pregnancy is unsafe and may lead to fetal deformities or intellectual disability (*n* = 712, 55.9%). In contrast, knowledge was lower for more specific or technical topics. Fewer than half of participants recognized the association between repeated radiation exposure and bone deformities (*n* = 534, 41.9%); only *n* = 459 (36.0%) correctly identified the approximate equivalence between a spiral CT scan and multiple conventional X‐ray images, and approximately 18% (*n* = 235) correctly identified that magnetic resonance imaging (MRI) does not use ionizing radiation.

Knowledge gaps were also observed for other long‐term health effects of radiation exposure. Approximately half of the participants correctly identified that repeated exposure may cause clouding of the eye lens (cataracts) (*n* = 652, 51.2%) and delayed growth in children (*n* = 678, 53.2%). Fewer than half were aware that therapeutic and diagnostic radioactive materials lose radioactivity over time due to decay (*n* = 516, 40.5%). Just over half of participants recognized the role of pharmacists in educating patients about the safety of therapeutic radioactive materials (*n* = 725, 56.9%). The median knowledge score was 5 (IQR: 3–7) out of a maximum possible score of 10. The internal consistency of the knowledge composite was acceptable, with a Cronbach's alpha of 0.74.

Regarding attitudes toward radiation, most participants expressed concern about repeated exposure to radiological imaging. Trust in physicians' decision‐making was also high, with the majority agreeing that radiologic examinations are requested only when necessary. Participants reported positive attitudes toward professional education, particularly the involvement of pharmacists in radiation safety counselling, and expressed a willingness to participate in radiation safety awareness sessions if available. In contrast, perceptions of the adequacy and clarity of warning signs in radiology departments were mixed, with a substantial proportion of participants reporting neutral or negative views (Table S2).

The attitude composite had a Cronbach's alpha of 0.51, indicating limited internal consistency. This suggests that the items may reflect heterogeneous dimensions of radiation‐related attitudes rather than a single unidimensional construct. In terms of practices and information‐seeking behaviour, 846 participants (66.4%) reported reading warning signs in radiology departments, whereas 428 (33.6%) had not. Fewer than half, 561 participants (44.0%), perceived these signs as sufficient and useful, while 713 (56.0%) did not. Notably, 772 respondents (60.6%) reported never having asked a healthcare provider about radiation safety before undergoing imaging. Among the full sample, 254 participants (19.9%) reported consulting a medical physicist or radiographer, 207 (16.2%) a physician, 26 (2.0%) a nurse, and 15 (1.2%) a pharmacist. Self‐rated knowledge followed a broad distribution, with 901 participants selecting values between 40% and 80% (Figure 3).

**FIGURE 3:**
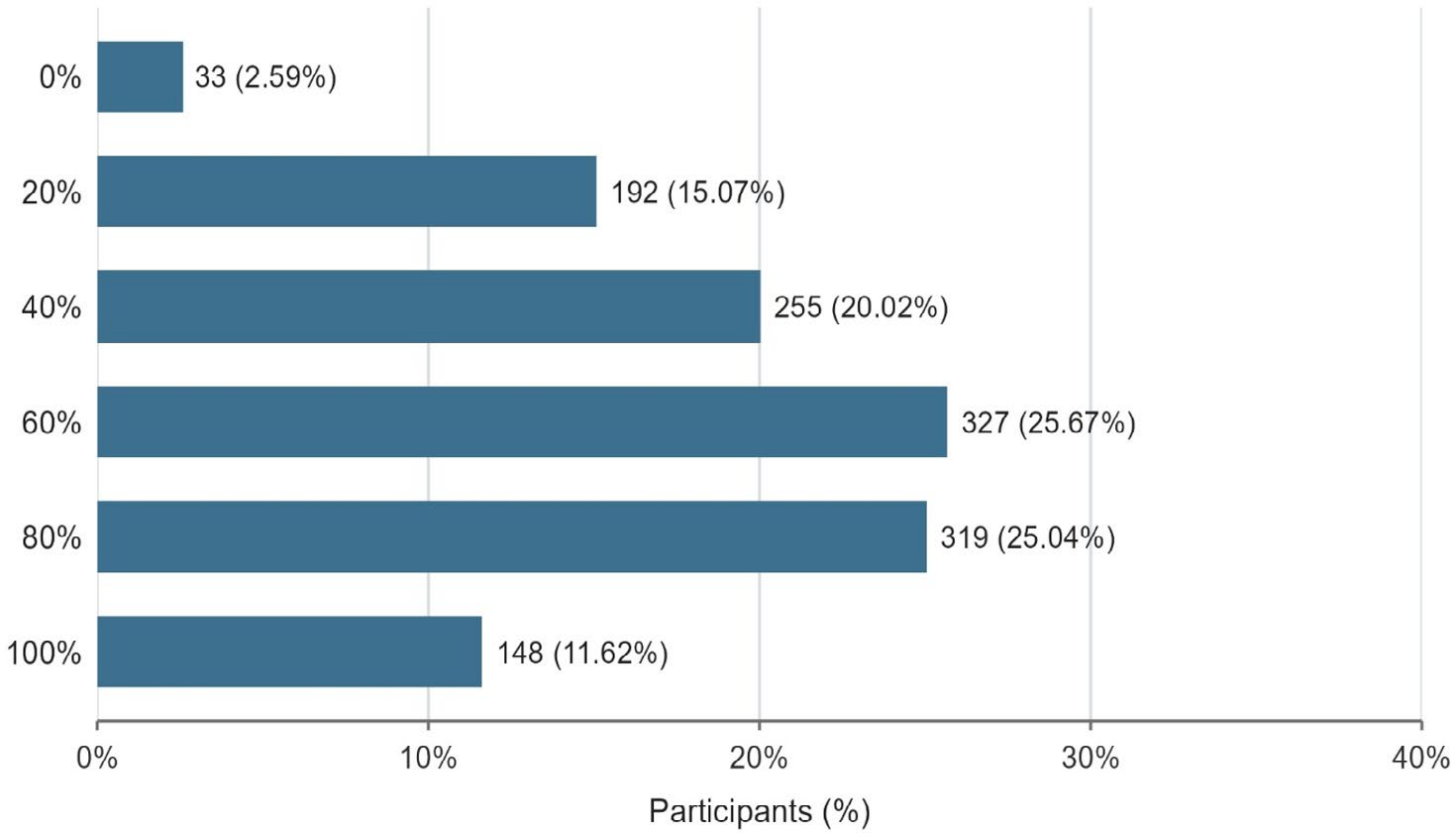
Participants**'** self‐rated knowledge of radiation risks, reported in 20‐percentage‐point categories among surveyed adults in Iraq (*N* = 1,274).

Nearly half of participants reported no prior exposure to radiation risk awareness sessions or educational materials (*n* = 632, 49.61%). Among those who had received information, lectures were the most commonly reported source (*n* = 282, 22.14%), followed by social media platforms (*n* = 166, 13.03%) and YouTube (*n* = 144, 11.30%). In contrast, consultations with healthcare professionals were rarely reported (*n* = 28, 2.20%), and only a small proportion of participants relied on published online articles (*n* = 22, 1.73%) (Figure 4).

**FIGURE 4:**
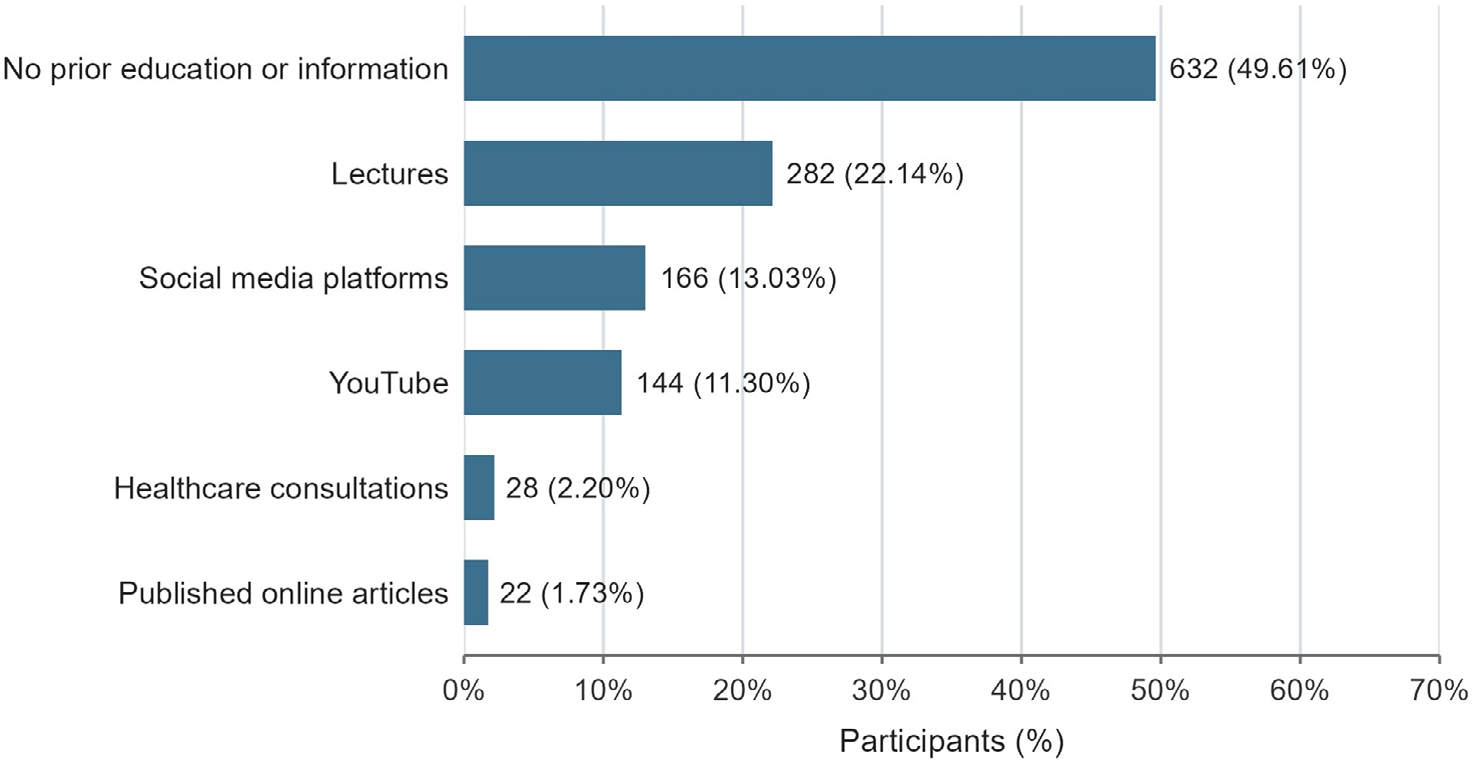
Previous exposure to radiation‐risk education or information sources among surveyed adults in Iraq (*N* = 1,274). Bars show the reported primary source of radiation‐risk information.

Table 2 presents the median quantile regression analysis examining factors associated with radiation‐related knowledge scores after excluding attitude score from the model. None of the examined demographic, educational, work/study‐field, residence, occupational‐exposure, or recent‐exposure variables was significantly associated with the conditional median knowledge score. Compared with participants aged > 40 years, those aged 18–30 years had an estimated one‐point higher median knowledge score, but the association was not statistically significant (β = 1.000, 95% CI: −0.699 to 2.699, *p* = 0.248); no difference was observed for those aged 31–40 years (β = 0.000, 95% CI: −1.662 to 1.662, *p* = 1.000). Compared with postgraduate education, the estimated coefficients were −1.000 for primary/secondary education or less (95% CI: −3.043 to 1.043, *p* = 0.337), −1.503 for a diploma (95% CI: −3.501 to 0.495, *p* = 0.140), and −1.000 for a bachelor's degree (95% CI: −2.472 to 0.472, *p* = 0.183); none was statistically significant.

**TABLE 2:** Quantile regression analysis of factors associated with radiation‐related knowledge score.

Parameter	Category/comparison	β	*p*	95% CI lower	95% CI upper
Intercept		6.000	**< 0.001**	4.117	7.883
Gender	Female	0.000	1.000	−1.022	1.022
	*Male (reference)*	0	—	—	—
Age	18–30 years	1.000	0.248	−0.699	2.699
	31–40 years	0.000	1.000	−1.662	1.662
	*> 40 years (reference)*	0	—	—	—
Educational level	Primary/secondary school or less	−1.000	0.337	−3.043	1.043
	Diploma	−1.503	0.140	−3.501	0.495
	Bachelor's degree	−1.000	0.183	−2.472	0.472
	*Postgraduate degree (reference)*	0	—	—	—
Work/study field	Radiation‐related field	0.000	1.000	−1.300	1.300
	Medical field	0.000	1.000	−1.202	1.202
	*Non‐medical field (reference)*	0	—	—	—
Area of residence	Rural	0.000	1.000	−1.055	1.055
	*Urban (reference)*	0	—	—	—
Continuous occupational radiation exposure	Yes	1.000	0.330	−1.012	3.012
	No	−1.000	0.124	−2.274	0.274
	*Maybe (reference)*	0	—	—	—
Direct radiation exposure during the previous six months	1–5 times	0.000	1.000	−1.098	1.098
	> 5 times	0.000	1.000	−2.164	2.164
	*No exposure (reference)*	0	—	—	—

*Note:* Median quantile regression was performed at *q* = 0.50. Reference categories were male gender, age > 40 years, postgraduate education, a non‐medical work/study field, urban residence, ‘maybe’ for continuous occupational radiation exposure, and no direct radiation exposure during the previous six months. Two‐sided *p* values < 0.05 are shown in bold.

Abbreviations: β, regression coefficient; CI, confidence interval.

Continuous occupational radiation exposure was also not significantly associated with knowledge. Compared with participants who were unsure, the estimated coefficients were 1.000 for those reporting exposure (95% CI: −1.012 to 3.012, *p* = 0.330) and −1.000 for those reporting no exposure (95% CI: −2.274 to 0.274, *p* = 0.124). Gender, work/study field, area of residence, and frequency of direct radiation exposure during the previous six months were likewise not significantly associated with knowledge.

Table 3 presents the quantile regression analysis examining factors associated with participants' attitudes toward radiation. Higher knowledge scores were significantly associated with higher median attitude scores (*β* = 0.114, 95% CI: 0.052 to 0.177, *p* < 0.001).

**TABLE 3:** Quantile regression analysis of factors associated with attitudes toward radiation.

Parameter	Category/comparison	β	*p*	95% CI lower	95% CI upper
Intercept		16.000	**< 0.001**	15.311	16.689
Knowledge score	Per one‐point increase	0.114	**< 0.001**	0.052	0.177
Gender	Female	0.357	**0.031**	0.032	0.682
	*Male (reference)*	0	—	—	—
Age	18–30 years	0.071	0.796	−0.470	0.613
	31–40 years	−0.100	0.710	−0.628	0.428
	*> 40 years (reference)*	0	—	—	—
Educational level	Primary/secondary school or less	0.443	0.183	−0.209	1.094
	Diploma	0.371	0.253	−0.266	1.009
	Bachelor's degree	−0.029	0.905	−0.498	0.441
	*Postgraduate degree (reference)*	0	—	—	—
Work/study field	Radiation‐related field	0.343	0.104	−0.070	0.756
	Medical field	−0.057	0.770	−0.440	0.326
	*Non‐medical field (reference)*	0	—	—	—
Area of residence	Rural	0.286	0.096	−0.050	0.622
	*Urban (reference)*	0	—	—	—
Continuous occupational radiation exposure	Yes	−0.537	0.103	−1.182	0.109
	No	0.029	0.890	−0.378	0.435
	*Maybe (reference)*	0	—	—	—
Direct radiation exposure during the previous six months	1–5 times	0.171	0.335	−0.177	0.520
	> 5 times	−0.329	0.349	−1.016	0.359
	*No exposure (reference)*	0	—	—	—

*Note:* Median quantile regression was performed at *q* = 0.50. Reference categories were male gender, age > 40 years, postgraduate education, a non‐medical work/study field, urban residence, ‘maybe’ for continuous occupational radiation exposure, and no direct radiation exposure during the previous six months. Two‐sided *p* values < 0.05 are shown in bold.

Abbreviations: β, regression coefficient; CI, confidence interval.

Female participants had higher median attitude scores than males (*β* = 0.357, 95% CI: 0.032 to 0.682, *p* = 0.031). Rural residence was not significantly associated with attitude score compared with urban residence (*β* = 0.286, 95% CI: −0.050 to 0.622, *p* = 0.096). No significant associations were observed between attitude score and age group, educational level, work/study field, occupational radiation exposure, or frequency of direct radiation exposure during the previous six months.

## 4 Discussion

This study assessed radiation‐related knowledge and attitudes among the general population in Iraq and identified factors associated with these outcomes. Participants recognized several major radiation risks but showed clear gaps in technical concepts, cumulative exposure, and the characteristics of different imaging modalities; attitudes were generally cautious and supportive of radiation‐safety education [27, 28]. These findings align with previous studies conducted in regional and international contexts, which have similarly reported incomplete public understanding of medical radiation despite widespread exposure to diagnostic imaging [8, 11, 12].

International comparisons indicate that the Iraqi pattern is not unique. In a multicentre Italian survey of 2866 patients, 57.0% did not recognise MRI as radiation‐free and 55.1% did not know that chest CT delivers a higher radiation dose than chest radiography [12]. A Hong Kong study likewise reported unsatisfactory awareness of radiation dose and risk, including frequent misconceptions regarding MRI and the relative dose associated with CT [27]. In the United Kingdom, only 22% of orthopaedic outpatients recognized that CT uses ionizing radiation associated with cancer risk, and only 25% correctly understood that CT carries greater radiation risk than plain radiography [29]. These findings closely parallel the modality‐specific gaps observed in Iraq and suggest that limited radiation literacy persists across different healthcare systems.

Although most participants correctly identified serious health risks associated with radiation, such as cancer and reproductive harm, knowledge was substantially lower for more technical or less intuitive concepts [30, 31]. In particular, limited awareness that magnetic resonance imaging does not use ionizing radiation and weak understanding of the relative radiation dose associated with computed tomography reflect persistent misconceptions about imaging modalities. Similar gaps have been reported in other patient and public surveys, with evidence showing that many individuals struggle to distinguish between ionizing and non‐ionizing imaging techniques and may incorrectly attribute radiation exposure to MRI [32, 33].

Misunderstanding of CT‐related radiation dose is especially important given that CT contributes disproportionately to population radiation exposure from medical imaging [2, 6]. Previous studies have shown that patients often lack an appropriate frame of reference for interpreting radiation dose and have difficulty comparing exposure across imaging modalities when information is not clearly contextualised [29, 34]. As a result, individuals may either overestimate risk and experience unnecessary anxiety or underestimate cumulative exposure and accept repeated imaging without fully appreciating potential long‐term implications [35]. These findings demonstrate the importance of clear, standardised communication that explicitly differentiates between imaging modalities and presents radiation risk in a way that supports informed decision‐making [13].

Knowledge related to long‐term and delayed effects of radiation exposure was also inconsistent. While approximately half of participants recognized risks such as cataract formation and impaired growth in children, fewer were aware that radioactive materials lose radioactivity over time through decay. This pattern suggests that public understanding of radiation risk tends to prioritise outcomes that are either widely publicised or intuitively alarming, while cumulative, time‐dependent, or probabilistic effects receive less attention [35]. Similar patterns have been observed in other studies, where awareness of radiation risk is often dominated by associations with cancer, with substantially lower recognition of deterministic effects such as cataracts or growth‐related outcomes [12, 13].

Evidence from radiation protection research indicates that public discourse and patient education frequently emphasise carcinogenic risk, despite growing recognition that non‐cancer effects, including radiation‐induced cataracts, can occur at lower doses than previously assumed [5]. Likewise, limited understanding of radioactive decay has been reported in studies examining public and patient knowledge of diagnostic and therapeutic radiation, suggesting gaps in foundational concepts that underpin radiation safety messaging [36]. Together, these findings indicate that radiation risk communication often lacks balance, with insufficient emphasis on cumulative exposure, delayed effects, and basic radiation principles. Addressing these gaps may require educational approaches that move beyond cancer‐centric narratives and provide clearer explanations of long‐term and time‐dependent radiation effects in accessible terms [37, 38].

Attitudes toward radiation were generally cautious but not overtly negative. Most participants expressed concern about repeated exposure to imaging while also reporting high levels of trust in physicians' decisions to request radiologic examinations. This coexistence of concern and trust has been described in previous work, where patients rely heavily on clinician judgement even when they perceive potential risks associated with imaging [9, 13]. Such findings highlight the importance of clear and balanced communication from healthcare providers regarding both the benefits and risks of diagnostic imaging [38, 39].

Participants also demonstrated positive attitudes toward education on radiation safety and expressed a willingness to participate in awareness sessions if available. Support for the involvement of pharmacists in radiation safety counselling was high, despite pharmacists being rarely consulted in practice. This gap between perceived importance and actual engagement suggests missed opportunities for patient education within routine care [40, 41]. Similar mismatches have been reported elsewhere, where pharmacists and other allied health professionals are recognized as credible sources of information but remain underutilised in discussions about radiation risks and safety [10, 15]. Strengthening the visibility and integration of these roles may help address existing knowledge gaps.

The adjusted attitude model identified a positive cross‐sectional association between knowledge and attitude scores: Higher knowledge scores were associated with higher attitude scores. Attitude score was not included as a predictor in the knowledge model to avoid reciprocal adjustment between two concurrently measured outcomes. Accordingly, this finding should not be interpreted as evidence that greater knowledge causes higher attitude scores; it indicates only that the two measures were related after adjustment for the included demographic and exposure variables [9, 13].

None of the examined sociodemographic variables, including gender, age, educational level, work/study field, and residence, was independently associated with knowledge. Some previous studies have reported age‐related differences in radiation awareness [27, 31], whereas others have linked higher educational attainment with greater awareness [8, 12]. Variations in sampling, population composition, and measurement may partly explain the inconsistency. The present findings do not support targeting radiation‐safety education solely according to age or formal education; materials should remain accessible across levels of health literacy [42].

Continuous occupational radiation exposure was associated with knowledge in the unadjusted yes‐vs.‐no subgroup comparison, but neither the “yes” nor “no” category differed significantly from the “maybe/unsure” category in the adjusted knowledge model. This differs from prior research reporting greater radiation awareness among people working in radiation‐related environments [10, 15] and indicates that the bivariate contrast in this study should not be interpreted as an independent occupational effect. In the attitude model, female participants had higher scores than males, consistent with literature on gender differences in health‐risk perception [43], in which women often report greater concern about potential health risks [9].

Patterns of information‐seeking behaviour further contextualise these findings by highlighting gaps between knowledge needs and information provision. Although most participants reported reading warning signs in radiology departments, fewer perceived these signs as sufficient or useful. The majority had never asked a healthcare provider about radiation safety prior to imaging, and when information was sought, it was most often from radiographers or medical physicists rather than physicians or pharmacists [28, 44]. Similar reliance on passive information sources and limited patient–provider communication has been documented elsewhere, suggesting that radiation risk discussions are not routinely integrated into clinical encounters [12, 13]. This may reflect time constraints, assumptions about patient understanding, or uncertainty among clinicians about how best to communicate radiation risks [45, 46].

Nearly half of participants reported no prior exposure to radiation risk education, and among those who had received information, lectures and social media were the most common sources, while direct consultation with healthcare professionals was rare. This pattern suggests that communication about radiation risks remains largely informal, opportunistic, and inconsistent, rather than systematically embedded within routine care [44, 46]. Previous work has emphasised that effective radiation risk communication requires proactive, structured approaches that go beyond written signage or passive information delivery [13]. Given that participants expressed willingness to engage in educational initiatives, these findings point to clear opportunities for improving accessible, standardised radiation safety education across healthcare settings, particularly through direct patient–provider interactions and interdisciplinary involvement [14].

The limited direct communication observed in Iraq also resembles patterns reported elsewhere. At an Australian radiology clinic, 85.6% of patients stated that their referring physician had not provided information about radiation dose and risk before imaging, and only 34.2% correctly identified CT as involving a higher radiation dose than conventional X‐ray imaging [28]. In Finland, 95% of patients wished to receive information about radiation dose and associated risks, with the prescriber, an information letter, and the radiographer identified as preferred sources [37]. Similarly, in the Italian multicentre survey, 80.4% preferred to receive radiation information from healthcare professionals, whereas only 42.7% had been informed about radiation risks during an imaging examination [12]. These comparisons reinforce the international gap between patients' information needs and the communication routinely provided in clinical practice.

### 4.1 Strengths, Limitations, and Future Directions

Several strengths of this study should be highlighted. First, the study included a large sample of 1274 adults recruited from multiple regions of Iraq, substantially exceeding the calculated minimum sample size and permitting the adjusted examination of associations across demographic, educational, and radiation‐exposure characteristics. By focusing on the general adult population, the study also extends the available Iraqi evidence beyond research conducted predominantly among medical students and healthcare professionals. Second, the questionnaire underwent a structured process of content review and cultural adaptation that included evaluation by a multidisciplinary expert panel, independent forward and back translation, two rounds of cognitive pretesting, and separate technical pilot testing. These procedures strengthened the relevance, comprehensibility, and cultural appropriateness of the instrument for use among Iraqi adults. Third, the study examined several interconnected dimensions, including radiation‐related knowledge, attitudes, previous exposure experiences, information‐seeking practices, and communication with healthcare professionals, thereby providing a broader assessment of radiation literacy than studies limited to factual knowledge alone. Finally, the use of median quantile regression was appropriate for the bounded and non‐normally distributed outcome scores and enabled adjusted estimation of factors associated with both knowledge and attitudes without relying on normality assumptions.

Several limitations should be acknowledged. The cross‐sectional design precludes causal inference, while voluntary convenience sampling through social‐media platforms may have introduced self‐selection related to age, educational attainment, interest in health information, and digital literacy and may have reduced participation among individuals with limited internet access [47, 48]. However, limitations arising from non‐probability recruitment should be distinguished from the population coverage and administrative advantages afforded by online data collection. Internet use in Iraq reached approximately 81.5% of the population in 2024, providing broad potential access to an internet‐administered questionnaire [49]. The sex and residence distributions of the study sample were also broadly comparable with those reported in the 2024 Iraqi census. Women constituted 50.6% of the sample compared with approximately 49.8% nationally, while rural residents constituted 34.9% of the sample compared with approximately 29.8% nationally, indicating that urban residents were not overrepresented [50]. These similarities provide partial reassurance regarding sex and geographic coverage, although they do not establish representativeness across age, educational attainment, health literacy, or other measured and unmeasured characteristics. Online self‐administered questionnaires may also provide greater perceived privacy and reduce interviewer‐related social‐desirability effects; experimental evidence has demonstrated more accurate reporting of sensitive information through web administration than through conventional telephone interviewing [51]. Internet surveys can additionally reach geographically dispersed populations efficiently and at lower cost, and online samples may be demographically diverse; they can also generate population‐representative estimates [51, 52, 53]. Nevertheless, because the present study used voluntary non‐probability recruitment without population weighting, residual self‐selection cannot be excluded.

Although online self‐administration may reduce interviewer‐related social‐desirability effects, it does not eliminate recall error or the possibility that respondents provide answers they perceive as more acceptable. All information in this study was self‐reported and may therefore have been affected by recall and response biases. Participants may have had difficulty accurately recalling the timing, frequency, or type of previous imaging examinations, whether radiation risks or precautions were explained, and whether protective equipment was used. They may also have overstated safety‐conscious behaviours or selected responses they perceived as socially desirable. No clinical records or occupational‐exposure records were available to verify these responses [54].

An additional limitation concerns the measurement properties of the questionnaire. Formal construct validity, including structural, convergent, and discriminant validity, was not assessed. The knowledge and attitude totals were developed as content‐based composite indices rather than as reflective psychometric scales intended to measure a single latent trait or a prespecified multidimensional structure; accordingly, factor‐analytic assessment was not prespecified. Although expert content review, cross‐cultural adaptation, cognitive pretesting, and pilot testing supported the relevance, clarity, and cultural appropriateness of the items, these procedures do not establish construct validity. The total scores should therefore be interpreted as pragmatic summaries of the included items rather than as fully validated latent‐trait measures. In addition, the attitude composite demonstrated low internal consistency, which may partly reflect the inclusion of items addressing heterogeneous aspects of radiation‐related attitudes; nevertheless, further studies should undertake dedicated psychometric evaluation before the instrument is used to support latent‐trait or domain‐level inferences [54].

Despite these limitations, this study provides valuable insight into radiation‐related knowledge and attitudes within the Iraqi population. The findings identify specific and actionable knowledge gaps, particularly in relation to technical concepts, cumulative exposure, and differentiation between imaging modalities. Future research would benefit from longitudinal or interventional designs to examine how knowledge and attitudes evolve over time and to evaluate the effectiveness of targeted educational interventions. Further work is also needed to develop and validate more robust, multidimensional measures of attitudes toward radiation risk [55].

At a practice level, future research should move beyond describing knowledge gaps and focus on how radiation risk information is actually communicated in clinical settings. This includes examining when, by whom, and in what form discussions about radiation risks take place, as well as how patients understand and use this information when making decisions about imaging [38, 39]. Due to the limited engagement with healthcare professionals reported in this study, there is a clear need to evaluate interventions that embed radiation risk communication more explicitly into routine care, rather than relying on warning signage or passive information sources alone [45, 46]. Developing and testing educational approaches that are sensitive to differences in age, educational background, and health literacy may help improve patient understanding and engagement with radiation safety [42].

## 5 Conclusion

This study provides a comprehensive assessment of radiation‐related knowledge and attitudes among the general population in Iraq, highlighting both areas of awareness and persistent gaps. While participants generally recognized serious health risks associated with radiation exposure, understanding of more technical concepts, cumulative exposure, and modality‐specific differences remained limited. Attitudes toward radiation were largely cautious but accompanied by high trust in clinicians' decisions, emphasising the central role of healthcare providers in shaping patient perceptions and engagement.

The adjusted knowledge model did not identify independent demographic, educational, work/study‐field, residence, or radiation‐exposure predictors of knowledge. In the attitude model, higher knowledge scores and female gender were associated with higher attitude scores. Patterns of information‐seeking revealed limited direct communication with healthcare professionals and heavy reliance on passive or informal sources, pointing to missed opportunities for patient education within routine care. Together, these results suggest that improving radiation literacy requires more than general awareness raising. Integrating clear, consistent, and accessible radiation‐risk communication into everyday clinical interactions, supported by interdisciplinary involvement, may help address the gaps identified and promote more informed decision‐making around medical imaging.

## Funding

The authors have nothing to report.

## Ethics Statement

Ethical approval for this study was granted by the Department of Pharmacy at Al‐Rasheed University College (reference number: RUCPD10012604). Electronic informed consent was obtained from all participants prior to questionnaire completion.

## Conflicts of Interest

The authors declare no conflicts of interest.

## Supporting information


**Table S1:** Knowledge of Radiation‐Related Risks.
**Table S2:** Participants' Attitudes Toward Radiation.

## Data Availability

The data that support the findings of this study are available from the corresponding author upon reasonable request.
