# Supplementary material for: Radiation‐Related Knowledge and Attitudes Toward Medical Imaging Among the General Population in Iraq: A Cross‐Sectional Survey
Source: J Med Radiat Sci. 2026 Sep 30:10.1002/jmrs.70123. Online ahead of print. doi: 10.1002/jmrs.70123 (PMC13625136; doi:10.1002/jmrs.70123)
Supplement: Supplementary file 1 [file JMRS-9999-0-s001.docx]

**Table S1. Knowledge of Radiation-Related Risks**

|  | **Incorrect** | **Correct** |
| --- | --- | --- |
| Exposure to radiation during pregnancy is safe and does not cause deformities or intellectual disability to the fetus. | 562 (44.1%) | 712 (55.9%) |
| Does the MRI machine use ionizing radiation? | 1039 (81.6%) | 235 (18.4%) |
| Radiation may cause cancer. | 243 (19.1%) | 1031 (80.9%) |
| Repeated exposure to radiation may cause clouding of the eye lens (cataract). | 622 (48.8%) | 652 (51.2%) |
| Repeated exposure to radiation may cause bone deformities. | 740 (58.1%) | 534 (41.9%) |
| High doses of radiation may cause infertility (temporary or permanent). | 452 (35.5%) | 822 (64.5%) |
| Repeated exposure to radiation may cause delayed growth in children in the long term. | 596 (46.8%) | 678 (53.2%) |
| A spiral CT scan (helical CT) is equivalent to 100 to 800 regular X-ray images. | 815 (64%) | 459 (36%) |
| Therapeutic and diagnostic radioactive materials may lose their radioactivity over time due to decay. | 758 (59.5%) | 516 (40.5%) |
| A pharmacist can play an important role in educating patients about the safety of therapeutic radioactive materials. | 549 (43.1%) | 725 (56.9%) |

**Note:** Items 1 and 2 were reverse coded.

**Table S2. Participants' Attitudes Toward Radiation**

|  | **Strongly disagree** | **Disagree** | **Neutral** | **Agree** | **Strongly agree** |
| --- | --- | --- | --- | --- | --- |
| I feel worried about repeated exposure to X-rays or imaging (CT scan). | 20 (1.6%) | 43 (3.4%) | 250 (19.6%) | 482 (37.8%) | 479 (37.6%) |
| I trust that doctors request radiologic examinations only when necessary. | 42 (3.3%) | 93 (7.3%) | 229 (18%) | 565 (44.3%) | 345 (27.1%) |
| It is important for pharmacists to participate in educating patients about the risks and benefits of radiation and radioactive materials. | 24 (1.9%) | 77 (6%) | 268 (21%) | 511 (40.1%) | 394 (30.9%) |
| Warning signs in radiology departments are clear and sufficient. | 75 (5.9%) | 252 (19.8%) | 344 (27%) | 431 (33.8%) | 172 (13.5%) |
| I would participate in an awareness session about radiation safety if available. | 27 (2.1%) | 70 (5.5%) | 271 (21.3%) | 557 (43.7%) | 349 (27.4%) |

**Note:** The first item was reverse coded.
